# Physical activity in Brazil: lessons from ELSA-Brasil. Narrative review: Atividade física no Brasil: lições do ELSA-Brasil. Revisão narrativa

**DOI:** 10.1590/1516-3180.2017.0023190317

**Published:** 2017-07-31

**Authors:** Francisco José Gondim Pitanga, Maria Conceição Chagas Almeida, Ciro Oliveira Queiroz, Estela Maria Leão de Aquino, Sheila Maria Alvim Matos

**Affiliations:** I PhD. Associate Professor, Department of Physical Education, School of Education, Universidade Federal da Bahia (UFBA), Salvador (BA), Brazil.; II PhD. Public Health Researcher, Gonçalo Moniz Institute, Fundação Oswaldo Cruz (FIOCRUZ), Salvador (BA), Brazil.; III MSc. Assistant Professor, Escola Bahiana de Medicina e Saúde Pública (EBMSP), Salvador (BA), Brazil.; IV MD, PhD. Full Professor, Instituto de Saúde Coletiva, Universidade Federal da Bahia (UFBA), Salvador (BA), Brazil.; V PhD. Associate Professor, Instituto de Saúde Coletiva, Universidade Federal da Bahia (UFBA), Salvador (BA), Brazil.

**Keywords:** Exercise, Social determinants of health, Epidemiology, Epidemiologic factors, Hypertension, Diabetes mellitus

## Abstract

**CONTEXT AND OBJECTIVE:**

The Brazilian Longitudinal Study of Adult Health (ELSA-Brasil) was conducted among civil servants at six higher education institutions located in six Brazilian state capitals. The objective of this review was to identify the publications produced within the scope of ELSA-Brasil that analyzed the participants’ physical activity.

**DESIGN AND SETTING:**

Review study using baseline data from ELSA-Brasil.

**METHODS:**

Narrative review of Brazilian studies on physical activity produced using data from ELSA-Brasil participants.

**RESULTS:**

The prevalence of leisure-time physical activity (LTPA) among ELSA-Brasil participants was low (44.1% among men and 33.8% among women). The main factors associated were social (higher schooling and family income), environmental (living in places with conditions and opportunities for physical activity) and individual (not being obese, being retired, not smoking and positive perception of body image). The perception of facilities for walking in the neighborhood was positively associated with both LTPA and commuting-related physical activity. An active lifestyle was a protective factor against several cardiometabolic variables (hypertension, diabetes, lipid abnormalities and cardiovascular risk over the next 10 years). Comparison between LTPA and commuting-related physical activity showed that only LTPA had a protective effect against arterial hypertension.

**CONCLUSIONS:**

The prevalence of physical activity among ELSA-Brasil participants was low. The main determinants were social, environmental and personal. LTPA had a greater protective effect on cardiometabolic outcomes than did commuting-related physical activity.

## RESUMO

**CONTEXTO E OBJETIVO:**

O Estudo Longitudinal de Saúde do Adulto (ELSA-Brasil) é um estudo longitudinal com servidores públicos de seis instituições de nível superior localizadas em seis capitais brasileiras. O objetivo desta revisão foi identificar as publicações realizadas no âmbito do ELSA-Brasil que tenham analisado a atividade física dos participantes.

**TIPO DE ESTUDO E LOCAL:**

Estudo de revisão com dados da linha de base do ELSA-Brasil.

**MÉTODOS:**

Revisão narrativa dos estudos sobre atividade física no Brasil produzidos com dados de participantes do ELSA-Brasil.

**RESULTADOS:**

A prevalência da atividade física no tempo livre (AFTL) em participantes do ELSA-Brasil foi baixa, (44,1% em homens e 33,8% em mulheres). Os principais fatores associados foram de ordem social (maior escolaridade e renda familiar), ambiental (viver em locais com condições e oportunidades para prática de atividade física) e individual (não ser obeso, ser aposentado/a, não ser tabagista, e ter percepção positiva da imagem corporal). A percepção de facilidades para caminhar na vizinhança foi positivamente associada tanto a AFTL quanto a atividade física no deslocamento (AFD). O estilo de vida ativo fisicamente foi fator de proteção para diversas variáveis cardiometabólicas (hipertensão arterial, diabetes, alterações lipídicas e risco cardiovascular nos próximos 10 anos). Após comparação entre AFTL e AFD, observou-se que apenas a AFTL apresenta efeito protetor para hipertensão arterial.

**CONCLUSÃO:**

A prevalência da atividade física em participantes do ELSA-Brasil foi baixa, os principais determinantes foram de ordem social, ambiental e pessoal. A AFTL apresentou maior efeito de proteção para desfechos cardiometabólicos do que a AFD.

## INTRODUCTION

At the beginnings of humankind, during the prehistoric period, people depended on their physical strength and ability to survive. They were nomads and, in their constant migrations in search of food and shelter, they made long walks along which they fought, ran and jumped. Thus, they were extremely physically active.^1^ Over the centuries, humans have undergone progressive reduction in their levels of physical activity, which was accentuated by the industrial revolution and more forcefully by the current technological revolution, although this reduction varies according to culture and social class. Currently, physical activity is defined as any body movement produced by the skeletal muscles that results in energy expenditure above the levels of resting metabolism.^2^ It is contextualized into four domains: leisure time, work, commuting and household-related physical activity.

For the Brazilian Longitudinal Study of Adult Health (ELSA-Brasil), it was decided to include only the domains of leisure time and commuting in the data collection. Physical activity at work and household activities were not recommended for assessment because of a tendency to overestimate the results from these domains in Latin America.^3^ In addition, these domains not included in ELSA-Brasil have only shown slight associations with possible health benefits.^4^^,^^5^^,^^6^

Epidemiological studies on physical activity can be categorized in accordance with the model for population studies on physical activity and health proposed by Pitanga.^7^ In this model, physical activity can be investigated both as a dependent variable and as an independent variable. When used as a dependent variable, the prevalence of physical activity and associated factors in population groups is studied. As an independent variable, the main consequences of physical activity are analyzed in relation to different outcomes. In the case of ELSA-Brasil, these were mainly cardiometabolic outcomes.

## OBJECTIVE

The objective of this narrative review was to identify the epidemiological studies conducted within the scope of ELSA-Brasil that have analyzed the participants’ physical activity both as a dependent variable and as an independent variable.

## METHODS

This was a narrative review of Brazilian studies on physical activity produced using data from ELSA-Brasil participants.

### What is ELSA-Brasil?

ELSA-Brasil was a cohort study on 15,105 women and men between the ages of 35 and 74 who were active or retired civil servants. They were recruited at six higher education and research institutions located in the cities of Salvador, Vitória, Belo Horizonte, Rio de Janeiro, São Paulo and Porto Alegre. The methodological details of the study have been described previously.^8^^,^^9^

Briefly, the data analyzed in the articles included in the present review were obtained at the baseline of ELSA-Brasil, between 2008 and 2010. The data had been gathered by a team of interviewers and certifiers, who had been trained and certified by a quality control committee^9^ to implement the study protocol at any ELSA-Brasil Research Center. A questionnaire had been applied then by means of face-to-face interviews. The protocol for ELSA-Brasil was approved by research ethics committees at the six centers involved in the study.

### Assessment of physical activity

To identify and quantify physical activity, the International Physical Activity Questionnaire (IPAQ) was used in the Elsa-Brasil. This consists of questions relating to the frequency and duration of physical activities (walking and moderate or vigorous exercise) that are developed at work, in going from place to place (commuting), in domestic activities and during leisure time.^10^ In ELSA-Brasil, only the domains of leisure time and commuting were evaluated. Physical activity was measured in minutes/week by multiplying the duration of each of the activities performed by the respective weekly frequency. The prevalence of leisure-time physical activity (LTPA) stratified according to some variables analyzed in ELSA-Brasil is presented in Table 1.

**Table 1:** Prevalence of leisure-time physical activity (LTPA), stratified according to the variables analyzed in the Longitudinal Study of Adult Health (ELSA-Brasil), 2008-2010^13^

Variables	Men		Women	
Prevalence of LTPA	6788	44.1 (42.9-45.3)	8088	33.8 (32.8-34.8)
Age (years)				
34-50	3168	44.9 (42.3-47.6)	3750	31.4 (28.7-34.1)
51-59	2102	40.2 (37.1-43.8)	2619	33.5 (30.4-36.7)
≥ 60	1518	47.6 (43.8-51.2)	1719	39.7 (36.0-43.5)
Education				
Incomplete elementary	564	27.8 (21.2-35.7)	326	19.0 (10.4-31.4)
Complete elementary	569	34.4 (28.5-41.8)	444	20.7 (12.9-30.4)
High school	2221	39.8 (36.6-43.1)	2918	25.8 (22.7-29.1)
College	3434	51.2 (48.8-53.6)	4400	41.6 (39.3-43.9)
Monthly family income				
Up to 2 MW	79	32.9 (17.2-55.7)	112	19.6 (5.2-40.3)
2 MW to 8 MW	2654	35.8 (32.8-38.9)	3217	24.9 (21.9-28.0)
8 MW to 18 MW	2238	46.0 (42.9-49.1)	3069	34.7 (31.8-37.6)
Above 18 MW	1791	54.8 (51.6-57.9)	1654	50.9 (47.5-54.4)
Suitable conditions for physical activity				
No	1706	36.1 (31.3-39.0)	2281	26.0 (22.5-2973)
Yes	4703	47.5 (45.4-49.5)	5390	37.6 (35.5-39.8)
Opportunities for physical activity				
No	1659	35.5 (31.6-39.5)	1971	22.9 (19.2-27.2)
Yes	4911	47.7 (45.7-49.8)	5903	37.8 (35.8-39.9)
Functional status				
Active	5698	43.0 (41.0-45.0)	6239	31.7 (29.6-33.4)
Retired	1087	49.9 (45.6-54.2)	1844	41.3 (37.7-44.9)

MW = minimum wage.

## RESULTS

Up to the present time, six papers using ELSA-Brasil baseline data that allow reflection on the main characteristics of physical activity in this population have been published. These papers deal with leisure-time and commuting-related physical activity both as dependent variables and as independent variables. In addition, another study compares the effects of leisure-time physical activity with those from commuting, with hypertension as the outcome.

### Physical activity as the outcome (prevalence and associated factors)

The first study on leisure-time physical activity published within the scope of ELSA-Brasil aimed to identify the associations of body image and obesity with physical activity, and considered 13,286 participants aged 35-64 years. The main results showed that body image dissatisfaction was less likely associated with moderate physical activity among women and of vigorous physical activity among men. It was also observed that men and women with central obesity and total obesity were less likely to engage in both high and moderate-intensity physical activity. Furthermore, overweight men were more likely to engage in vigorous physical activity.^11^

Subsequently, the associations between the perceived characteristics of the neighborhood and physical activity were explored. This was a cross-sectional analysis on 14,749 ELSA-Brasil participants and the associations were tested through multinomial logistic regression. The main results observed were that the perception that the neighborhood was more walkable was positively associated with reports of participation in leisure-time physical activity, and with greater likelihood of practicing this for a longer time during the week. The perception that the neighborhood was more walkable increased the likelihood of practicing physical activity for more than 150 min/week or up to 150 min/week (in comparison with no physical activity). The perception that the neighborhood was more accessible for walking was also positively associated with active commuting (Table 2).^12^

**Table 2:** Association between perceived walkability and physical activity. ELSA-Brasil, 2008-2010^12^

Variables	Better walkability
Leisure-time physical activity	
< 150	1.40 (1.28-1.52)
≥ 150	1.69 (1.57-1.83)
Commuting-related physical activity	
< 150	1.08 (0.99-1.17)
≥ 150	1.19 (1.09-1.30)

Next, the prevalence and factors associated with leisure-time physical activity were identified. A hierarchical ecological model was built with the possible factors associated with LTPA grouped into blocks. Odds ratios (ORs) and 95% confidence intervals (95% CIs) were estimated using logistic regression. The prevalence of LTPA among the ELSA-Brasil participants was 44.1% among men and 33.8% among women. Among men, having a higher education level, having a higher family income, living in environments with conditions and opportunities for physical activity, being retired and being overweight were positively associated with LTPA, while current smoking, obesity and abdominal obesity were negatively associated. Among women, being over 60 years old, having a higher education level, having a higher family income, living in an environment with conditions and opportunities for physical activity and being retired were positively associated with LTPA, while being overweight or obese and having abdominal obesity were negatively associated.^13^

### Physical activity as independent variable (cardiometabolic consequences)

In Brazil, studies on the main cardiometabolic consequences of physical activity are very important because they provide specific interpretations for the Brazilian population. Much of the existing information has been based on studies carried out abroad.

The first study published within the scope of ELSA-Brasil with physical activity as an independent variable had the main objective of identifying the association of leisure-time physical activity with cardiometabolic health. This study was developed with 10,585 participants aged 35-74, without cardiovascular diseases. Leisure-time physical activity status was defined using the American Heart Association and World Health Organization recommendations (≥ 150 min/week of moderate activity or 75 min/week of vigorous activity). After adjusting for confounding factors, the positive effects of leisure-time physical activity on cardiometabolic parameters were evident. The main results demonstrated an inverse association between leisure-time physical activity and arterial hypertension, diabetes and cardiovascular risk over the next 10 years, among both men and women^14^ (Table 3).

**Table 3:** Association between physical activity and cardiometabolic outcomes. ELSA-Brasil, 2008-2010^14^^,^^16^

Variables	Leisure-time physical activity	Commuting-related physical activity
Hypertension		
Men	0.75 (0.65-0.87)	No associations
Women	0.78 (0.66-0.92)	1.11 (1.01-1.21)
Diabetes		
Men	0.73 (0.61-0.87)	Data unavailable
Women	0.83 (0.67-1.03)	Data unavailable
Cardiovascular diseases over next 10 years		
Men	0.67 (0.57-0.78)	Data unavailable
Women	0.78 (0.65-0.93)	Data unavailable

Subsequently, a more recent paper aimed to assess the association of leisure-time physical activity intensity and duration with HDL-C, LDL-C and triglyceride levels. This was a cross-sectional study on 12,688 participants who were not on lipid-lowering medication. After adjusting for confounding factors, multiple linear regression was used to evaluate the association of intensity and duration of leisure-time physical activity with HDL-C, LDL-C and triglyceride levels. Both moderate and vigorous physical activity were found to be significantly associated with higher levels of HDL-C and lower levels of triglycerides. There were no significant associations of leisure-time physical activity with LDL-C levels.^15^

### Associations of leisure-time and commuting physical activity with cardiometabolic outcomes

Finally, associations of leisure-time physical activity and commuting-related physical activity with high blood pressure were investigated among ELSA-Brasil participants. Hypertension was defined as systolic/diastolic blood pressure of > 140/90 mmHg or use of antihypertensive medications. Out of the total samples of 15,105 participants, 13,857 subjects without previous cardiovascular diseases were analyzed. The association between physical activity and hypertension was determined using Poisson regression with adjustment for confounding variables. The results showed that among both men and women, leisure-time physical activity was inversely associated with hypertension. However, in relation to commuting, the association with hypertension was positive among women and without statistical significance among men^16^ (Table 3).

### Final remarks

One possible limitation of this study is that the information on physical activity was obtained through self-reporting questionnaires. Nevertheless, these instruments are widely used in national and international studies. 

The results presented here are examples of the potential for analysis of physical activity within ELSA-Brasil. This also includes the possibility that, in the near future, longitudinal data analyses may become feasible. Measurements of greater objectivity, such as accelerometry, can be expected to become incorporated, which may increase the validity of information on physical activity.

## CONCLUSIONS

Through these results, it can be stated that the prevalence of leisure-time physical activity was low among ELSA-Brasil participants. This is probably reproduced in similar populations, especially those involved in academic work, and it deserves to be studied in Brazil.

The results show that different variables within the social environment (greater schooling and family income) physical setting (living in places with conditions and opportunities for physical activity) and individual sphere (obesity, being retired and smoking) present associations with physical activity. Through these results, it can also be inferred that body image and nutritional status present relationships with physical activity, for both sexes, but that the nature of the association with physical activity differs according to sex. It can also be stated that perceived walkability was independently associated with the practice of physical activity, both during leisure time and in relation to commuting.

Regarding the consequences of physical activity practices, the results confirm that leisure time physical activity was a protective factor against cardiometabolic disorders in the ELSA-Brasil cohort, both for men and for women, whereas active commuting did not have any cross-sectional association with cardiometabolic outcomes. 

It can be suggested that incentive programs promoting physical activity practices should be implemented in populations similar to ELSA-Brasil that work in academic environments and are exposed to a sedentary daily life, considering that physically active behavior is a protective factor against different metabolic and cardiovascular diseases.
