# ERRATUM

**DOI:** 10.1590/1516-3180.2017.0023190317errata

**Published:** 2017-08-21

In the paper “Physical activity in Brazil: lessons from ELSA-Brasil. Narrative review”, with DOI number 10.1590/1516-3180.2017.0023190317, published in the Sao Paulo Medical Journal, volume 135, issue number 4, pages 391-5, on page 391:

Where it read:

“^IV^MD, PhD. Full Professor, Institute of Public Health, Universidade Federal da Bahia (UFBA), Salvador (BA), Brazil.”

It should read:

“^IV^MD, PhD. Full Professor, Instituto de Saúde Coletiva, Universidade Federal da Bahia (UFBA), Salvador (BA), Brazil.”

